# Social media shaping autism perception and identity

**DOI:** 10.1177/13623613241230454

**Published:** 2024-02-22

**Authors:** Ingjerd Skafle, Elia Gabarron, Anders Nordahl-Hansen

**Affiliations:** 1 Østfold University College, Norway; 2 University of Oslo, Norway; 3 Norwegian Centre for E-Health Research, Norway

**Keywords:** autistic adults, autistic communities, autistic identity, information-seeking behaviour, reflexive thematic analysis, social media

## Abstract

The aim of this study was to explore how autistic adults experienced using social media to find information about autism, and how they experienced online autistic communities. There is little research on the rationale why autistic people look for information on autism via social media, and on how such information is perceived from an autistic viewpoint. Twelve Norwegian autistic adults (aged 18–49 years) completed semi-structured interviews where they discussed social media and content specifically about autism and online autistic communities. Using reflexive thematic analysis, three main themes were developed. The themes were (1) Representation and Identity: An Online Journey; (2) An Unreliable, but Necessary Tool; and (3) Tensions and Discord. The findings indicated that even though social media was looked upon as an unreliable information source, the participants found information that helped them to learn about their experiences which had not been fully captured by the majority of the current research literature and online health sites. Also, social media groups for autistic people did not necessarily create a sense of community, as participants reported feeling alienated by hostile discussions. This study highlights the importance of having a greater variation and availability of information about autism online through official health channels.

**Lay abstract:**

This study suggested that social media can provide important information about autism to autistic people. We interviewed 12 autistic adults (aged 18–49 years) and talked to them about the use of social media to find both general information and content specifically about autism, autism identity and online autistic communities. There is little research exploring how autistic people find information about autism on social media and how that makes them feel. Therefore, it is important to ask autistic people about their experiences with using social media to obtain content about autism. The 12 participants explained that when they searched for information about autism on the official health pages, they often felt that the information they found was insufficient and could not answer their questions. In addition, they searched on social media platforms for information about autism despite that they perceived social media as an unreliable source. On the social media platforms, many found content that was positive in relation to their autistic identities. The participants also found comfort in some of the forums and social media groups and received helpful advice. Nevertheless, some of the discussions were aggressive and the participants felt alienated, which did not provide a sense of community online. The findings from the study may advice on what is missing in the official pages about autism, and highlight the need to involve the autistic community in writing the content on such platforms.

The Internet has been a catalyst for the autistic community, enabling them to unite across borders and overcome the communication challenges often faced by autistic individuals (Alper, 2023). Autistic people diverge from neurotypical social and communication patterns which can make face-to-face interactions in larger groups challenging and exhausting (Davis et al., 2022). Online forums and social media platforms have emerged as a valuable social engagement alternative for many autistic individuals (Bagatell, 2010; Wayman, 2021). Social media platforms have also become a common source of information, and increasingly play a crucial role in news consumption (Matsa, 2023). In 2008, during the early onset of social media, Davidson (2008) professed that the Internet would become a force for the autistic community, given its communication platform options.

Autistic culture, a relatively recent phenomenon, has predominantly flourished on the Internet, closely intertwined with the burgeoning neurodiversity movement (Kapp, 2020). In 1996, the first self-hosted autistic community, known as Independent Living (InLv), was established (Singer, 2017). Over the subsequent decades, there has been a remarkable surge in awareness driven by the voices of autistic people themselves. Consequently, it is crucial to understand which role social media plays on how information about autism is being disseminated and communicated, as well as its influence on the perception of autism both within the general public and among the autistic community.

Previous research has explored social media in relation to autism from different perspectives. For example, parents of autistic children and adolescents use online forums or social media to find support and health information about autism (Ahmed et al., 2019; Carter, 2009; Clifford & Minnes, 2013; Cole et al., 2017; Loukisas & Papoudi, 2016; Reinke & Solheim, 2015). Abel et al. (2019) examined 500 Facebook groups with 905,655 English-speaking users and their stated purposes. These Facebook groups were first and foremost designed to give support to autistic people and their family members. The majority of the groups were made up of parents and families (57.4%) and autistic people (23.4%). Bellon-Harn et al. (2020) investigated online depiction of autism by analysing 100 of the most viewed YouTube videos in which content about autism was directed to families of children with autism. A vast majority of the content focused on signs and symptoms.

Some studies have suggested that autistic children and youths spend less time on social media than their neurotypical peers and spend more time on gaming or non-social online activities (Alhujaili et al., 2022; Begara Iglesias et al., 2019; Mazurek et al., 2012; Mazurek & Wenstrup, 2013). While research involving autistic adults indicates that they might prefer to use social media for interacting with others, they also use social media at times in a qualitatively different way than neurotypical peers. Mashat et al. (2016) interviewed 12 Saudi Arabian autistic adults about their social media habits. The study showed a wide range of use of social media depending on the participants’ functioning level. Participants who lived more independently reported that they used social media platforms for chatting, connecting with friends and for educational purposes (Mashat et al., 2016). In a survey study by Gillespie-Lynch et al. (2014), 291 US autistic adults and 311 non-autistic adults were recruited. The results showed that the autistic adults enjoyed meeting new people with similar interests and were less preoccupied with using social media to keep in touch with friends and family compared to the non-autistic participants. In addition, aspects such as expressing one’s true self, and being part of discussion groups based on one’s identities, were reported to be more beneficial and joyful by the autistic study group than the non-autistic control group (Gillespie-Lynch et al., 2014).

Studies that focus on autism, social media use, friendship, or quality of social life diverge in outcomes when it comes to benefits and limitations. In a study by Mazurek (2013) of 108 US autistic adults who submitted a questionnaire about their social media use and friendship status, 79.6% of the sample reported using social media. A majority of the participants (64.9%) reported that they used social media for social purposes, whereas 22.1% said that they used social media for entertainment such as gaming or seeking out information based on strong interest. In addition, those who used social media were more likely to report having close friends. Moreover, greater offline friendship quality and quantity, and not social media use, were associated with decreased loneliness. Ward et al. (2018) studied social media use and happiness in 106 autistic US adults using an online survey. Happiness was measured using a four-item 7-point scale called ‘subjective happiness scale’, and the results suggested that Facebook use specifically could increase well-being. In a study by van Schalkwyk et al. (2017), the researchers assessed social media use, anxiety and friendship quality in 44 US autistic adolescents, and 56 clinical comparison controls. Social media use was significantly associated with higher friendship quality in autistic adolescents, which was moderated by the adolescents’ anxiety levels.

Social media can provide a space where autistic people can explore identities and not worry about hiding their autistic traits (Jedrzejewska & Dewey, 2022; Kelly et al., 2022). Kelly et al. (2022) undertook a systematic review of the lived experiences of 50 autistic women from nine qualitative studies. The women had been diagnosed late in adulthood. The study showed that following their diagnosis, these autistic women used social media to contact other autistic people and developed neurodivergent-affirming autistic identities. Jedrzejewska and Dewey (2022) explored camouflaging behaviour in 40 British autistic and 158 non-autistic adolescents. Their findings indicated that autistic adolescents camouflaged less online than in the offline context compared to non-autistic adolescents. ‘Camouflaging’ is a set of strategies that include hiding behaviours associated with autism to prevent others from seeing social difficulties (Hull et al., 2017).

Even though online communication might be benficial for some autistic people, there are still some risks involved. Triantafyllopoulou et al. (2022) investigated cyberbullying, cyber-aggression and self-esteem in 78 European autistic adults. The results suggested that high levels of social media use was associated with an increased risk of cyberbullying and also correlated negatively with self-esteem due to a higher propensity for being ignored on network cites and chatrooms. On the other hand, feelings of belonging to an online community were positively correlated to self-esteem. In a systematic review by Hassrick et al. (2021) of how autistic people use internet and social media to communicate, the authors found 32 eligible studies, and of these, 23 were studies of autistic adults. The results showed that one of the benefits of social media for autistic people includes more control over how they talk and engage with others online and a greater sense of calm during interactions. However, findings suggest some drawbacks, including continued feelings of loneliness and the desire for in-person friendships. However, social media also provided opportunities to find other autistic peers and form a stronger identity as part of an autistic community.

There is some research on how autism is perceived and represented in social media. YouTube, for example, offers the possibility for self-representation (Brownlow et al., 2013). Angulo-Jiménez and DeThorne (2019) analysed the representation of autism in 39 YouTube videos authored by self-identified autistic individuals. These videos were posted for the most part by American White male adults who reported having Asperger’s syndrome. An interesting result from this study was that 51% of the vloggers presented a positive take on autism, meaning that they highlighted what was positive about their autistic traits. A recent study examined the reach and accuracy of the most viewed TikTok videos (*n* = 133) about informal autism information (Aragon-Guevara et al., 2023). The researchers found that 27% of the videos were classified as accurate, while 41% were classified as inaccurate and 32% as overgeneralized. Alper et al. (2023) analysed 89 TikTok videos with common autism hashtags (e.g. #actuallyautistic) and 847 user comments. Their analyses suggest there is a complex relationship between autistic individuals and social media algorithms in defining what it means to be autistic, but also that such videos can inform mental health issues for autistic people. The researchers also suggest that self-diagnosis may be more accepted on TikTok than on other social media platforms where autistic groups are formed more on basis of a formal diagnosis. Egner (2022) studied how autistic people use social media to build individual and collective narratives via Twitter. She applied a qualitative virtual ethnographic approach, and collected data through data scrapes from Twitter. Her findings suggest that social media is used to construct a more positive group identity for autistic people that counter stereotypical and inaccurate representations. She claims that other media representations such as TV series and films about autistic people are ‘oversimplified, highly medicalized, rely on stereotypical depictions, and present autism as a series of deficits’ (p. 356). A study by Nordahl-Hansen et al. (2018) supports this notion. They assessed whether portrayals of autistic characters in 26 film and TV series aligned with *Diagnostic and Statistical Manual of Mental Disorders* (5th ed.; DSM-5) diagnostic criteria. The results showed that the autistic TV representations typically aligned so well with all DSM-5 criteria that it was unrealistic. Other studies have suggested that TV and film portrayals of autistic people are not perceived as authentic by many in the autistic community, and that there is a need for greater diversity in autistic characters (Dean & Nordahl-Hansen, 2022; Orm et al., 2023). It can be hypothesized that such lack of authentic autistic portrayals makes autistic people search for representation on social media. There is, however, little research on autism representation and portrayals on social media. In addition, a lot of the research on autism and social media focus on adolescents. It is therefore imperative to explore how autistic adults experience online interactions, information-seeking and how these aspects influence their identity and perception of autism.

## Purpose of this study

The aim of this study was to explore how autistic adults experienced using social media to find information about autism, and how they experienced online autistic communities. By interviewing autistic adults, we explore social media use from an autistic perspective.

The following research questions (RQs) guide this study:

*RQ1*. What are autistic adults’ perspectives on social media’s contribution to building an autistic identity?
*RQ2*. What are autistic adults’ perspectives on social media’s potential role in building an autistic community?
*RQ3*. How do autistic adults experience social media as a source of information related to autism?

## Method

### Design

We chose a qualitative design to gather the perspectives of autistic adults. Semi-structured interviews were chosen to gather in-depth insight, and the interviews were later transcribed and analysed using reflexive thematic analysis (TA; Braun & Clarke, 2022). We have conducted this study in accordance with the ICMJE Recommendations for the Protection of Research Participants (International Committee of Medical Journal Editors, 2023). The Norwegian Agency for Shared Services in Education and Research (SIKT) approved this study (Ref. 446745). Although the questions asked in this study were not deemed sensitive, the fact that the participants shared their diagnosis was considered to be sensitive information. Therefore, data from the interviews are stored in the highly secure platform named Services for sensitive data, TSD.

### Participants

We used a purposive sampling strategy, but also snowballing when recruiting the participants. Eleven participants were recruited through posts on three open Facebook groups for autistic people: ‘Autism parents in Norway’, ‘Parents and family who have autistic children, youths, and adults surrounding them’ and ‘Autism, ADHD, Tourette’s – spectrum’. Permission to post was obtained from the administrators of the groups. We also contacted the Norwegian Autism Association, which sent out requests to their members via email. One participant was recruited using other contacts. To participate, one needed to be 18 years old or older, be autistic and be a user of any type of social media. In Norway, the diagnostic manual from the World Health Organization (WHO, 1993) The *ICD-10 International Classification of Mental and Behavioural Disorders* (10th ed.; ICD-10) is still employed, and as a result, the participants had an autism diagnosis based on ICD-10 criteria. We did not ask for any medical record validating the diagnosis, meaning that we potentially did not exclude self-identified autistic people. Table 1 shows the participants’ demographic information at a group level to maintain anonymity.

**Table 1.** Participant demographics (*N* = 12).

	*N* or *M* (*SD*) [range]
Gender	
Man	5
Woman	6
Non-binary	1
Ethnicity	
Norwegian	12
Age (years)	32.6 (10.06) [18–49]
Age at diagnosis (years)	23.5 (16.02) [3–49]
Autism diagnosis	
Asperger’s	10
PDD-NOS	1
Childhood autism	1
Highest education level	
Postgraduate	4
Undergraduate	2
High school graduate	3
12th grade no diploma	3
Employment status	
Student	4
Unemployed	4
Full-time employment	4

PDD-NOS: pervasive developmental disorder-not otherwise specified.

### Interviews

The first author travelled to different locations in Norway to meet and interview the participants using a semi-structured interview approach (Braun & Clarke, 2013; Crabtree & DiCicco-Bloom, 2006). The interviews were conducted between January and March 2023 and recorded. The participants had been offered to see the interview guide before the meeting, and some wanted to see the questions beforehand. The interviews lasted on average approximately 1 h, the shortest being 40 min and the longest 1 h and 20 min. The interviews were done in Norwegian and recorded with a dictaphone. The 12 participants signed written consents where they were promised that their personal identifiers would be permanently removed, and that the recordings would be deleted after completion of the study. They could withdraw from the interview at any given time.

### Reflexive TA

To analyse the data, the framework from reflexive TA was applied (Braun & Clarke, 2022). The steps taken in this analytical approach are flexible, interpretative and subjective. After the transcription of the recorded interviews was finalized by the first author, the transcripts were uploaded in the programme HyperRESEARCH 4.5.4. (Researchware, 2015), a tool used for qualitative research when it comes to coding, organizing and analysing qualitative data. In reflexive TA, the codes capture meanings within the dataset based upon the RQ(s). The first author did the coding. A single coder is considered good practice in reflexive TA (Braun & Clarke, 2022). The coding process was inductive, having in mind that this part of the process is deeply embedded in the researcher’s theoretical and social position. There are two types of codes in TA, semantic and latent. The former stays close to the participants’ language and explicit meaning, whereas the latter has a more conceptual and abstract level of meaning (Braun & Clarke, 2022). Coding in reflexive TA ranges from semantic to latent and can be combined, as in this study. The first author familiarized herself with the data several times through interviews, transcribing, coding and in the end analysing patterns of meaning to create the themes. The data extract of participant quotations used in this article was translated from Norwegian into English by the first author.

With respect to researcher positionality, no one in the research team identify as neurodivergent. Our academic backgrounds are from special education and psychology. In this study, we adopted an inductive orientation to data and an experimental orientation of reflexive TA (Braun & Clarke, 2022). The philosophical assumptions that underpin this study are critical realism ontology evolved by Bhaskar (1975). In critical realism, the real world is independent of our knowledge, but at the same time socially constructed by both language and context (Lopez & Potter, 2005). Therefore, the data in our study are not a clear reflection of reality, but reflections of each participant’s culture, language and life history which shape the way they perceive the social practice that social media is.

### Community involvement

The interview guide was developed with the help and consultation from an autistic adult who received financial compensation (see interview guide in Appendix 1). The consultant also read through the draft of the introduction with the literature review and pointed out some places where person-first language had been used. She also identified a question in the interview guide that would be difficult to answer because of the wording.

### Findings and analysis

The aim of this study was to explore how autistic adults experienced using social media to find information about autism, and how they experienced online autistic communities. Three subordinate themes were developed through TA: ‘Representation and Identity: An Online Journey’; ‘An Unreliable but Necessary Tool’; and ‘Tensions and Discord’. Below each main theme and subtheme, participant quotes are listed first, and subsequent analysis thereof. The themes are discussed to consider how this study relates to and extends existing knowledge. The quotes are reported together with participant code and gender.

Twelve autistic adults participated in the study. Six women, one non-binary and five men, participated. They had a mean age of 32.6 years (range = 18–49, *SD* = 10.06). In Norway, the diagnostic manual ICD-10 (WHO, 1993) is still used, so the participants had an autism diagnosis according to ICD-10. All participants identified themselves as White Norwegians. It is well recognized that the Norwegian Ministry of Health and Care Services is actively engaged in the implementation of the 11th edition of the *International Statistical Classification of Diseases and Related Health Problems (ICD-11)* (WHO, 2019), aligning with the *Diagnostic and Statistical Manual of Mental Disorders (5th ed; DSM-5)* (American Psychiatric Association, 2013). Consequently, within the Norwegian context, the terms ‘autism’, ‘autistic’ and ‘ASD’ are widely employed. There exists a general awareness and understanding of the modifications introduced by DSM-5 and ICD-11. Moreover, participants were recruited by specifically seeking autistic individuals in social media groups that used the word ‘autism’ and not any of the sub-diagnoses in ICD-10. Hence, the interpretation was that the participants considered themselves to be autistic. Table 2 gives an overview of the participants’ preferred social media platforms.

**Table 2.** Participants’ use of social media platforms.

Participant code	Social media platforms
1	Facebook, TikTok, Snapchat
2	Facebook, Instagram, LinkedIn
3	Facebook, Instagram, YouTube
4	TikTok, Facebook
5	Facebook
6	Facebook, Snapchat, Instagram, YouTube, TikTok
7	YouTube, Instagram
8	Facebook, Snapchat, YouTube, Twitter, Instagram
9	Facebook, Snapchat, Instagram
10	Instagram, Reddit, Twitter, Facebook
11	Facebook, Instagram, Snapchat
12	Facebook, WhatsApp, Discord, LINE, Snapchat, Instagram

After analysing the codes, three main themes and four subthemes were developed. Table 3 below shows examples of how the themes were generated.

**Table 3.** Reflexive thematic analysis.

Theme	Subthemes	Code label	Example data extracts
A: Identity and Representation: An Online Journey *Entails seeing or chatting with other autistic people that one identifies with. Social media provides a great variety of autistic people who embraces being autistic, something that is positive for self-esteem*	A1: A Positive Autistic Identity	Autistic role models	‘I have gained more self-esteem by listening to others who actually has it. They become role models in a way. I think life without social media would have been different. I would probably be fine, but in a different way. I think my self-image would have been different, and in a bad way because I notice that I get a lot of self-esteem and a good self-image by talking to other autistic people about their experiences’ (P1, W).
		A positive journey via social media	‘Yes, it has been positive! With all those YouTube videos and things that are shared . . . and especially right after I received my diagnosis . . . all I could think about was Rainman. Then I though, do I know of anyone else autistic? Are there any actors or famous people? I could not think of anyone’ (P3, W).
	A2: Finding Representation	Found female representation	‘It has given me insight into the female representation of Asperger and autism, and what the female symptoms are. Because a lot of the literature is based on men’ (P4, W).
		Recognizing oneself in others	‘I was watching these YouTube videos, and there was one who . . . he was very . . . well, he reminded me of myself in a way. I could see that he had the same turmoil inside’ (P 12, M).
Theme	Subthemes	Code label	Example data extracts
B: An Unreliable but Necessary Tool *Entails notions that there are a lot of misinformation about autism on social media, but despite this, social media provides an alternative place to gather necessary information lacking in official public health sites*	B1: Official Sites and Information Gaps	Nowhere else to go	‘I don’t want to find information on social media, because people post so many strange things, and I also feel as if I can’t trust it either. But in the end, that’s where I end up because I can’t find answers anywhere else’ (P11, W).
		Stigma in public health information	‘When I read on Norwegian Institute for Public Health’s pages, I think: “Oh my god, is that what people think of me?” I feel that that is where I meet most stigma – facing the public sector’ (P7, N-B).
	B2: Misinformation and Nuance	A nuanced picture of autism	‘I think it helps me, and it nuances it a lot. You have one point of view about autism from yourself. Then the parents have their view about their child, and then you have society’s view on the diagnosis. You can see all of that on social media, so it opens up a discussion between different types of interest groups’ (P4, W).
		Misinformation and information	‘In an average Facebook group for parents of autistic children, I think there’s 50-50 information and misinformation, and a majority of it is completely nonsense unfortunately’ (P3, W).
Theme		Code label	Example data extracts
C: Tensions and Discord *Entails notions of tensions and discord in some of the social media groups for autistic people. Although there is a great appreciation of many such social media groups when it comes to giving each other valuable support and advice, some noted tensions and harsh debates to be alienating at times.*		Polarizing debates	‘It is what I have mentioned earlier -the debates often get skewed. Because there are some who are very much in to one particular thing. It could be EIBI, or identity, or which word to use when talking about autism. And those people are often in charge of the debates’ (P5, M).
		Wrong words	‘For example, when in some of these groups there’s a young mother who says “Hi, my daughter was just diagnosed, and now that I understand she has autism, I’m wondering . . .” And then the first thing that comes up is criticism such as “don’t say that she has autism, you have to say autistic,” and then you wind up being afraid of saying something wrong. You become afraid of using the wrong words’ (P3, W).

EIBI: Early Intensive Behavioural Intervention.

### Theme A: Identity and Representation: An Online Journey

#### Subtheme A1: Positive Autistic Identity

Participants reported that seeing other autistic persons sharing content about their lives and experiences gave them a more positive self-image and better self-esteem. This came from both seeing or chatting with different autistic people and seeing that they were not ashamed of their diagnosis: ‘I think I would have had a much worse self-image, because I notice that I get a lot of self-esteem and a better self-image by seeing other autists who share their experiences’ (P1, W). Participant 3 (W) reported that social media content was overwhelmingly positive for her self-esteem: ‘I would say that it perhaps is 80–95% positive because you get a sense of a community and you don’t feel so alone, and you get more focus on the positives’. Others reported that they learnt more about autism and themselves through following autistic people on social media: ‘Yes, some of these groups have helped me a lot, especially the English ones. Some of them have pictures where things are explained more graphically, which is very helpful, for example about “masking” or whatever it is called’ (P2, M). Another participant reported that,

I think it has made me change my perspective on it, and now I give myself more slack. If I understand that I need to be alone, I put on my headphones and I no longer stop myself from doing it, because I know that I need it (. . .) there are a lot of things that I always have done, and ways I have behaved, that all of a sudden make sense. So, in that way it has sort of changed how I look at myself. (P10, W)

Participants talked about finding acceptance of being diagnosed as autistic through social media: ‘I do feel that the information I have gathered through social media, has made me come to terms with my own autistic person. So, it has been positive, because when you see other persons of my age talk about themselves, it is easier to identify’ (P4, W).

I have begun to search on social media and have seen a lot of people there saying ‘I’m autistic’ and ‘I’m autistic’ and ‘I’m autistic’, then you see that they can be just like me. It is not only like Raiman where you think ‘I don’t want to be like that’, or you don’t want to be like how they describe it on NIPH*, or in those old articles. So, I think it has something to do with identifying with someone which is really important. (P3, W)

*Norwegian Institute for Public Health

Participant 6 (W) also talked about gaining acceptance due to social media content about autism: ‘Yes, there was grief in the beginning, but now I have realized that I am born with the brain that I have been born with. It is just the way I am, and I am who I am’. The groups and forums also provided a safe space: ‘In those groups I feel safer in way. There are others there who are themselves, and we are a bunch of weirdos’ (P12, M).

#### Subtheme A2: Finding Representation

Many of the autistic participants reported that they missed having someone they could identify with, and that they lacked information and content where they felt represented. This was especially important for the women. They had found content in social media platforms that gave them a sought-after representation: ‘There are others that have felt what I have felt and had the same thoughts and challenges. It gives you recognition and the feeling that you are not alone in this’ (P4, W). This was especially true for platforms such as TikTok and YouTube. Participants 6, 3 and 4 elaborated on this topic:

When I think about what Asperger’s is, I think about a boy or a man. Very monotonous. Into trains. All that. And very strange. On Netflix, there’s this show – ‘Love on the Spectrum’. I don’t recognize myself in any of those people. It is very stereotypical. I have found more people I can relate to on YouTube. Girls . . . women . . . (P6, W)

I saw an Australian YouTube video (. . .) about a woman, at my age, who talks about . . . she talks about it in such a nice way . . . when you read the articles it is all about ‘those who have autism are like this and that, and can come across as impolite’, whereas she talks about herself which makes it easier to identify with, and she talks about her autism diagnosis and that she would not have been who she is without it, and I remember the first time I saw the video the tears just started to roll, because it was so nice to see a completely ordinary person talk about the same struggles, and she seemed to have no issue in doing so (P3, W).

Social media has brought me insight into the female representation of Asperger and autism, and what the female symptoms are. So much of the literature is based on men. (P4, W)

Participant 11 (W) said she would have felt more alone without having seen other autistic people on social media: ‘A new world opens up when you start to search in all those forums and groups that are on Facebook, and all those people who post on Instagram about their own diagnosis’. Participant 12 (M) reported that he became more confident about his own diagnosis after seeing a video of a man with autism on TikTok and YouTube: ‘When I see him, I think . . . I would not have assumed that he has autism, or something like that, only by looking at him. There was something there that made me believe that I also could be autistic’. Some participants explained that it could be educational to watch other autistic people share from their everyday life: ‘There are many people that I follow on Instagram who post about how it is for them, and then you get an insight into their experiences. And you learn quite a lot from that’ (P10, W).

Analysis of ‘Representation and Identity: An Online Journey’. Participants reported that seeing autistic people on social media talking openly about their diagnosis gave them a more positive perspective on autism. As seen in previous findings by Angulo-Jiménez and DeThorne (2019), YouTube videos by autistic users tend to have a positive perspective of autism and focus on the strengths of having autism. Many of the women in this study expressed that they missed information about autistic women, and that it was important to have someone to identify with. From the research that has been published so far, there is a lot of focus on boys and men, as historically, a lot more men have been diagnosed with autism. Autism is diagnosed 3 times more often in men than in women (Loomes et al., 2017). In recent times, there has been more focus on how autism in women is represented, and that autistic girls and adolescents, and women tend to camouflage more and thus making it harder to both detect and diagnose (Hull et al., 2017). In fact, autistic girls tend to receive their diagnosis at a much later stage than boys (Begeer et al., 2013). This study highlights the importance of having representation both in research literature and in official health information, and how that can help in the process of accepting an autism diagnosis and developing a positive autistic identity. Embracing a positive autism identity might prevent mental health struggles, as previous research studies show that autistic adults experience high rates of co-occurring mental health conditions such as depression and anxiety (Hollocks et al., 2019). A study of autism content in TikTok videos by Alper et al. (2023) suggests that content shared on TikTok contributes to awareness about mental health and autism, potentially influencing the well-being of autistic individuals engaging with this content on the platform. Similarly, many of the participants in this study expressed appreciation for autism content on social media, especially seeing other autistic people, and that this shaped their perception of autism and themselves in a positive way.

### Theme B: An Unreliable but Necessary Tool

#### Subtheme B1: Official Sites and Information Gaps

Participants reported both frustration and resignation about the information that they could find in official public health sites online or in books: ‘When I read on Norwegian Institute for Public Health’s pages, I think: “Oh my god, is that what people think of me?” I feel that that is where I meet most stigma – facing the public sector’ (P7, N-B). The content seemed outdated and provided little help for everyday challenges for autistic adults:

When it comes to autism identity and stuff like that, I would say I have learnt 80% via social media. Because there are very few books . . . in Norwegian there’s actually «nada» written after 1979 (. . .) In Norway . . . you will not find a lot. There’s HelseNorge and NIPH* and much of it is just random. The same goes for The Norwegian Autism Association. (P3, W)

*Norwegian Institute for Public Health

I have gained a lot of knowledge about what it is and how other people experience their sensory issues on YouTube and TikTok. What do I recognize? How do other people facilitate for their issues? I would have never gained that knowledge in a book. (P6, W)

Social media gives a more nuanced picture. But I am more concerned about changing the system to see what works, because it will make a difference for us with autism. It matters what health workers read, and for them it is not acceptable to find information on social media. They are supposed to gather information from official sources. (P7, N-B)

The participants had used official sites to find information about autism, but had noticed that a lot of the sites focused on children, and the way that both the language and the information were formulated was often perceived as alienating:

There’s so little out there, and that’s why I go to Facebook afterwards, because what I read online is just . . . it’s always about children . . .’ autistic children struggle with this’ . . . as if these children never grow up . . . as if they grow out of their autism. It is as if they just want to get these children through school, but what about family or working life? Why not write ‘autistic people struggle with . . .’ But If you search for information on Facebook and social media, it is more polarized then if you go to a more objective source. So that is what I really want. I always try that first, but even the Autism Association has a focus on children. (P11, W)

In the beginning, I read a lot, but the language is very dry and stiff . . . a sort of ‘legal language’. So, it gets harder to digest what it really says. The things you see on YouTube or TikTok are more vernacular, of people who are like yourself, and who describes how it is experienced. And you also have the option to contact them. (P12, M)

The participants wanted to find information on the official health pages, but after not having success in finding such information and despite knowing the shortcomings with the information posted on social media, they ended up searching social media platforms. This is elaborated in the next subtheme.

#### Subtheme B2: Misinformation and Nuance

The participants all expressed concerns about using social media as a source of information because of the lack of neutral and objective information. However, as the participants felt that they did not get the answers they needed in the official sources, they sought out additional information on social media:

I don’t want to find information on social media, because people post so many strange things, and I also feel as if I can’t trust it either. But in the end, that’s where I end up because I can’t find answers anywhere else. (P11, W)

Another participant, who used social media to find information about autism, also mentioned that there is a lot of misinformation on social media: ‘In an average Facebook group for parents of autistic children, I think there’s 50-50 information and misinformation, and a majority of it is completely nonsense unfortunately’ (P3, W). Participant 4 (W) addressed the same issue:

There’s a lot of information and misinformation, but a lot of it are experiences, and you can’t really say that it’s wrong. It is that person’s experience. Because of my special interest, which is in the academic field, I tend to look at sources from a very critical thinking point of view. A lot of the videos from TikTok address features of autism or ADHD. But a lot of psychologists have pointed out that the things they mention are very superficial. It does not go to the core, and it is created in a way that almost anyone can recognize themselves in it.

Participant 9 (M) said he used social media as a sort of backup:

First, I go to a webpage, to perhaps find out who wrote it. Then I find the name, and see ‘oh, they’re members there’. So, I use it as a substitute. So, official webpages and professionals are my main sources, and then social media is my spare wheel. You know, your GP could probably give you a better answer.

Participant 4 (W) commended social media for, despite misinformation, providing a more encompassing picture of autism:

Yes, it helps me a lot, and it refines it a lot. You have one point of view of autism from yourself – from the self. Then the parents have their view about their child, and then you have society’s view on the diagnosis. You can see all these views about autism on social media, and this opens up a discussion between different types of interest groups.

Analysis of the main theme *an Unreliable but Necessary Tool*: Many of the participants reported that they had tried to find useful information on official public health sites. However, these sites did not provide them with the kind of information they were looking for. Much of the information found online revolved around children. Earlier research has discussed the disproportion of autism content online focusing mainly on childhood, often with parents or caregivers of autistic children in mind (Stevenson et al., 2011). Further, Akhtar and colleagues (2022) raised the point that even though more information was available for adults in certain domains since the Stevenson and colleagues’ study in 2011, the information applicable to adults could be interpreted as ‘infantilizing’ in several ways.

Second, the autistic women who participated criticized that a lot of the official sources were about autistic men, or written in a way that made it sound as if it were about men or boys. The lack of information about the experiences of autistic women and girls has been confirmed in recent literature and considered a barrier for getting a diagnosis for girls and women (Lockwood Estrin et al., 2021). Therefore, as many of the participants explained, they did not want to find information on social media due to the amount of misinformation, but they expressed that there was nowhere else to go. They were painfully aware of the amount of misinformation that can be found on social media, which had been confirmed by previous findings in a study by Wang et al. (2019). They undertook a systematic review of health-related misinformation spread on social media and found that social media play an increasingly larger role in propagating such. For example, misinformation about the MMR vaccine and autism is still being spread online. Similarly, Aragon-Guevara et al. (2023) found that only 27% of the most popular TikTok videos about autism portrayed autism accurately, whereas the remaining videos were deemed inaccurate or overgeneralized by the researchers.

Another issue that came up was the way the information in typical official health sites and autism association pages was written. For some of the participants, the wording was not good for their self-esteem. For example, two of the participants mentioned that they had read on such pages that autistic people could be blunt and come across as rude, and that made them feel self-conscious and doubt themselves. Previous research on media portrayals indicates that autistic people often feel that such representations are stereotypical and medicalized, and that they miss diversity (Egner, 2022; Nordahl-Hansen et al., 2018; Orm et al., 2023). One could hypothesize that there is the same need for diversity on how autism is portrayed and described in health literature and online official health pages.

### Theme C: Tensions and Discord

Although the participants reported many positive outcomes with social media use related to autism, both in terms of finding useful information and seeing other likeminded, tensions and discord in the groups were also something that emerged as a theme. Most of the participants appreciated having alternative platforms to find autism-related content but did not necessarily feel part of a larger community of autistic people or seek out friendship with other autistic people. Others did not find it natural to make contact online: ‘I don’t find it easy to make friendships through a screen. To me that is not natural’ (P8, M). Previous research has also shown that autistic people do not necessarily use social media first and foremost for social purposes, but rather for entertainment or finding information (Begara Iglesias et al., 2019; Gillespie-Lynch et al., 2014). When the participants sought advice from groups online, they found that many people wanted to help:

Yes, I think it is of big help to the able to ask questions in the groups. I struggle with something, and there are others who struggle with the same. You get a lot of answers, and it is very nice. (P11, W)

Nevertheless, participants also reported that in some groups, especially Facebook-groups, there were a quite strict set of rules that needed to be followed, and an aggressive tone especially towards person-first language and interventions such as Applied Behavior Analysis (ABA) and Early Intensive Behavioural Intervention (EIBI): ‘In some Facebook-groups you are not allowed to write anything positive about ABA’ (P5, M). Participant 3 (W) expressed frustration over the sort of ‘policing’ when it came to language use:

For example, when in some of these groups there’s a young mother who says ‘Hi, my daughter was just diagnosed, and now that I understand she has autism, I’m wondering . . .’ And then the first thing that comes up is criticism such as ‘don’t say that she has autism, you have to say autistic’, and then you wind up being afraid of saying something wrong. You become afraid of using the wrong words. (P3, W)

Participant 5 (M) was also concerned about this:

There were some people who asked if others preferred to use ‘autistic’ or ‘person with autism’. Most people prefer ‘autistic’. I argued that one should vary, because there are still autistic people who don’t like the word ‘autistic’. Especially the older generation. We have to make those considerations too. (. . .) Even though I identify as autistic, I don’t demand that everyone else does. I think this can be very destructive. To make this debate so sharp and unnecessarily stale. (P5, M)

Participant 2 (M) felt uneasy by some of the disagreements in the social media groups:

For example, one says that children should not be forced to make eye contact. Then another one says ‘no, it’s good that the children learn to make eye contact’, then another one writes ‘No, it’s horrible!’. Then it becomes a major conflict. I don’t like to see that they cannot find a middle ground.

Participant 3 (W) expressed resignation:

I’m less active now. It has something to do with . . . especially in the large groups, you get attacked so easily and at such great force. It comes down to two things. One, is which methods to use to help autistic people, and whether to use behavioural therapy or nothing. The other thing is the choice of words.

Analysis of the main theme *Tensions and Discord*. Consistent with previous findings (Egner, 2022; Jedrzejewska & Dewey, 2022; Kelly et al., 2022), many of the participants felt less lonely by seeing other autistic people and establishing connections to a wider autistic community via social media platforms. Also, participants reported that many members in some of the Facebook groups were eager to help and give advice. This study provides more insight into how social media, and especially social media groups, plays a part in ongoing discourses and controversies within the autistic community. There were two issues that were seen as particularly polarizing. First, discussion about interventions, particularly ABA (Baer et al., 1968) and EIBI (Reichow et al., 2012). The participants had different opinions about such interventions, but overall, they were concerned about the harsh tone in the discussions. They thought that it was both frustrating and alienating that the debates were so harsh and aggressive. In a recent study by Gabarron et al. (2023), they identified that ABA was the third most discussed topic in tweets containing the hashtag #ActuallyAutistic. EIBI and ABA remain one of the most common interventions for autistic children, and meta-analyses reveal some effects in different domains such as intellectual and adaptive measures for autistic individuals (Eckes et al., 2023; Sandbank et al., 2020). However, there are not enough rigorous methodological designs to give adequate collective evidence supporting the effectiveness of EIBI (Davis et al., 2022; Sandbank et al., 2020). The controversy surrounding ABA and EIBI in the autistic community has grown over the last decade. Some studies have reported both negative side effects and negative long-time impact (Anderson, 2023; McGill & Robinson, 2021).

The second issue was the use of identity-first language (e.g. autistic person) instead of person-first language (e.g. person with autism). Recent studies have showed that identity-first language is preferred in the autistic community (Kenny et al., 2016), and that the preference for identity-first language is associated with a stronger autism identity (Bury et al., 2022). Moreover, in a study by Taboas et al. (2023), 87% of the autistic adults said they preferred identity-first language. However, a recent Dutch study showed a preference for person-first language among 68.3% of the autistic adults (Buijsman et al., 2023). So, even though identity-first language has become the preferred language in the autistic community, it might be alienating for those autistic people who prefer otherwise, or for those feeling policed by others being told they use the wrong words.

Due to differences in age, experience and how long the participants had been using social media, there were most certainly different expectations regarding autism information and autistic online communities. Their expectations might run counter to the online exchange established by the people already in those communities. The use of identity-first language has historically been crucial for the autistic community in developing a positive identity (Kapp, 2020). In an influential essay ‘Why I dislike “person-first” language’, Jim Sinclair (1999) explained that person-first language removes autism from the person’s identity and makes it sound negative. Therefore, identity-first language shows a proud autistic identity.

Although some of the participants expressed frustration with the online groups regarding language use, most of them referred to themselves as autistic. However, two things can co-exist at the same time: Acknowledging the importance of identity-first language and possessing an understanding for others who use person-first language, either because of their lack of knowledge on the subject or because of personal preference. Similarly, one can, for example, see how participant 4 (W) expresses both great appreciation and frustration of different neurodiversity groups on Facebook: ‘I feel that it is very prejudiced. If you go into the groups that focus on neurodiversity, you can’t really criticise or think outside the box (P4, W)’. Then later, she expresses gratitude for neuroaffirmative social media content: ‘The English groups have more focus on experiences and challenges. It creates a much larger sense of community when you gather experiences than to focus on the medical aspects’.

#### Practical implications

Many of the participants did not find useful information about being an autistic adult on the official Norwegian sites. To provide useful information, the official sites could consider updating their content on autism or have a separate section that deals with adults only. The language should be written in a way that speaks to autistic people and not just about them. Therefore, one suggestion would be to bring autistic persons in for consultation when writing such pages. Another important issue was the women’s lack of representation in official sources. This should also be updated, as one knows more about the representation of autism in women now than from a few years back. This is also of major importance at the systemic level so that stakeholders such as health workers and teachers can be better equipped to recognize symptoms and avoid mislabelling women. As for the discord found in the autistic social media community regarding ABA and EIBI interventions and identity-first versus person-first language, these issues need to be addressed more openly as they carry an emotional charge for many.

#### Limitations

The names of the Facebook pages used for recruitment, such as ‘Autism Parents in Norway’, indicated that the groups were oriented towards neurotypical adults. This might imply that the participants recruited through these pages spent more time on neurotypical oriented social media. Thus, there may be a gap in the knowledge production, particularly related to online neuroaffirmative autistic communities. This gap could inform suggestions for further research that focus on online autistic communities. It is important to mention that the participants in this study had different experiences in terms of how long they had been active on social media, what kind of expectations they had to autistic online communities, and, for example, knowledge of the neurodiversity movement. In future research, one could separate between new and old users of social media, and users who have different knowledge of the neurodiversity movement. Further, some of the follow-up prompts used yes/no questions, which may have caused some bias, although the first author did her best to follow up the conversation if only a yes/no answer was given.

We did not ask for any formal proof or confirmation of the diagnosis to participate in this study. Thus, we did not exclude self-diagnosed autistic people, as we are aware and acknowledge the barriers some individuals (especially women and people of colour) face getting diagnosed (Tromans et al., 2021; Zener, 2019). In some studies, where interviews have been done online, researchers experienced that some participants faked being autistic perhaps for the financial benefit of it (Pellicano et al., 2023). However, in this study, no financial compensations were offered to participate. Instead, the participants took time away from jobs and family to be interviewed in person. Arguably, there is a smaller chance that such were the case in this study.

## Conclusion

This study investigated autistic adults’ experiences of autism content and autistic communities on social media platforms, with the use of a qualitative design. The findings showed that social media content about autism provided a nuanced and positive picture of autism, and even contributed to a more positive autistic identity. Specifically, many of the autistic women interviewees described that the online platforms helped them to understand their experiences which were not fully captured by the majority of the current research literature online and/or health sites. Although the participants viewed information on social media as unreliable, they could not find enough useful information elsewhere. They reported that they missed information about being an autistic adult and facing challenges related to work, education, family and raising children. The social media groups for autistic people did not necessarily create a sense of community, as some groups had a quite aggressive tone, especially when it came to issues such as methods of intervention and the use of identity-first versus person-first language. This study provides more insight into mechanisms behind the use of social media to find health information, and the complexity on how this influences autistic identity and the understanding of autism.

## Supplemental Material

sj-docx-1-aut-10.1177_13623613241230454 – Supplemental material for Social media shaping autism perception and identity Supplemental material, sj-docx-1-aut-10.1177_13623613241230454 for Social media shaping autism perception and identity by Ingjerd Skafle, Elia Gabarron and Anders Nordahl-Hansen in Autism
