# Supplementary material for: Social media shaping autism perception and identity
Source: Autism. 2024 Feb 22;28(10):2489–502. doi: 10.1177/13623613241230454 (PMC11487866; doi:10.1177/13623613241230454)
Supplement: sj-docx-1-aut-10.1177_13623613241230454 – Supplemental material for Social media shaping autism perception and identity [file sj-docx-1-aut-10.1177_13623613241230454.docx]

# INTERVIEW GUIDE

Thank you for agreeing to participate in this interview. There are no right or wrong answers to any of my questions, as I am only interested in your experience in using social media.

Please note that this guide represents the main themes to be discussed, and includes the various prompts that may be used (examples given for each question).

Before we begin, it would be nice if you could tell me a little bit about yourself.

1. **Online social media habits:** Can you tell me about your use of social media?

**Prompts:** Which social media platforms do you use? Do you use one more than another?

**Prompts**: Which social media platforms (if any) do you visit daily? Do you any favourites, and if so why?

**Prompts**: Have your social media habits changed, for example have you moved from one platform to the other?

**Prompts:** How much time do you on average use on social media on a daily basis?

1. **The use of social media as a source of information:** How would you describe social media as a source of information?

**Prompts:** Do you prefer to use social media platforms to find useful information, or do you have other sources?

**Prompts:** Which social media platforms do you find give you trustworthy information?

**Prompts:** Do you think it is difficult to detect whether you can trust information from social media?

**Prompts:** Do you feel that you have become more enlightened by social media?

**Prompts:** Do you think social media has made us all more enlightened?

1. **Social media and autism:** Do you use social media to find information about autism, and if so has this been educational and informative?

**Prompts:** Do you share information about autism on social media platforms?

**Prompts:** Has any of the information about autism that you have come across via social media changed the way you perceive autism, and if so – how?

**Prompt:** Do you prefer social media platforms when sharing important information about autism, or do you think such information is better shared in other ways?

**Prompts:** Has social media influenced the way you see yourself as an autistic person?

1. **Online discussions and information about autism:** Do you participate in online discussions about autism in social media? If so, which social media platforms do you use?

If not, do you pay attention to/follow online discussions about autism in social media?

Have these discussions influenced your own perception of autism?

**Prompts:** There are, for example, online debates about the causes of autism. Have you come across that?

**Prompts:** There is also an ongoing discussion between those who see autism as a medical condition and a disability and those who see it as a natural variation of human beings and a different way of being. Have you come across this debate on social media platforms?

**Prompts:** Have you come across debates about vaccines and autism on social media platforms?

**Prompts:** Have you come across misinformation about autism on social media?

**Prompts:** Have you come across useful information about autism on social media?

1. **Online autistic communities:** Have you made connection/made friends with other autistic individuals through social media?

**Prompts:** How has this influenced you?

**Prompts:** How has this influenced the way you see other autistic persons?

**Prompts:** has it changed the way you see yourself/changed your identity?

**Prompt:** How has social media influenced your social life?

**Prompts:** Does online information/discussions make you feel like a part of an autistic community?

Is there anything else that you would like to comment on or discuss?

Thank you very much for your time and the information you shared today!
